# Feline aminopeptidase N is not a functional receptor for avian infectious bronchitis virus

**DOI:** 10.1186/1743-422X-4-20

**Published:** 2007-02-26

**Authors:** Victor C Chu, Lisa J McElroy, Jed M Aronson, Trisha J Oura, Carole E Harbison, Beverley E Bauman, Gary R Whittaker

**Affiliations:** 1 Department of Microbiology and Immunology, Cornell University, Ithaca, NY 14853, USA

## Abstract

**Background:**

Coronaviruses are an important cause of infectious diseases in humans, including severe acute respiratory syndrome (SARS), and have the continued potential for emergence from animal species. A major factor in the host range of a coronavirus is its receptor utilization on host cells. In many cases, coronavirus-receptor interactions are well understood. However, a notable exception is the receptor utilization by group 3 coronaviruses, including avian infectious bronchitis virus (IBV). Feline aminopeptidase N (fAPN) serves as a functional receptor for most group 1 coronaviruses including feline infectious peritonitis virus (FIPV), canine coronavirus, transmissible gastroenteritis virus (TGEV), and human coronavirus 229E (HCoV-229E). A recent report has also suggested a role for fAPN during IBV entry *(Miguel B, Pharr GT, Wang C: The role of feline aminopeptidase N as a receptor for infectious bronchitis virus. Brief review. Arch Virol 2002, 147:2047–2056*.

**Results:**

Here we show that, whereas both transient transfection and constitutive expression of fAPN on BHK-21 cells can rescue FIPV and TGEV infection in non-permissive BHK cells, fAPN expression does not rescue infection by the prototype IBV strain Mass41. To account for the previous suggestion that fAPN could serve as an IBV receptor, we show that feline cells can be infected with the prototype strain of IBV (Mass 41), but with low susceptibility compared to primary chick kidney cells. We also show that BHK-21 cells are slightly susceptible to certain IBV strains, including Ark99, Ark_DPI, CA99, and Iowa97 (<0.01% efficiency), but this level of infection is not increased by fAPN expression.

**Conclusion:**

We conclude that fAPN is not a functional receptor for IBV, the identity of which is currently under investigation.

## Background

The family of *Coronaviridae *is composed of group 1–3 coronaviruses (CoVs) [1]. These viruses are able to infect human, canine, feline, murine, bovine, porcine, rat, and avian species. The etiological importance and zoonotic characteristics of coronaviruses have received much attention since the discovery of the newly emerged severe acute respiratory syndrome associated coronavirus (SARS-CoV) in 2003 [1,2]. Coronaviruses have a high frequency of viral genome recombination and polymerase infidelity, which may have contributed to the increase of viral pathogenesis, inter-species transmission, and tissue tropism [3-5]. In the case of SARS-CoV, its ancestral origin remains undetermined, but some evidence suggests that Chinese horseshoe bats may be the natural reservoirs, while Himalayan palm civets harbor and support inter-species transmission to humans [5,6]. Other examples of extended tissue tropisms can also be found in some group 2 CoVs. It is speculated that the acquisition of hemagglutinin esterase (HE) activity from influenza C virus gives rise to the ability of sialic acid recognition and the extended tissue tropism and pathogenesis for some group 2 CoVs [7-9]. Furthermore, bovine coronavirus (BCV) is thought to have jumped to human hosts, possibly by recombining with influenza C virus, thus giving rise to human coronavirus-OC43 (HCoV-OC43) around 1890 [4,8].

Receptor interaction between the virus and its host is the first step leading to a successful entry and productive replication. Viruses increase fitness by adapting to environmental pressure through mutation and recombination. In contrast to other families of viruses that utilize a universal receptor to gain entry into host cells, members in the coronavirus family use a variety of cellular proteins and/or co-factors. Group 1 CoVs – including human coronavirus-229E (HCoV-229E), feline infectious peritonitis virus (FIPV), transmissible gastroenteritis virus (TGEV) and canine coronavirus (CCV) – utilize human, feline, porcine, and canine aminopeptidase N (APN) as functional receptors during virus entry [10-13]. The only notable exception is HCoV-NL63, which utilizes angiotensin-converting enzyme 2 (ACE2). In group 2 CoV, mouse hepatitis virus (MHV) of group 2a and SARS-CoV of group 2b independently utilize carcinoembryonic antigen-cell adhesion molecule (CEACAM1) and ACE2 to mediate infection [14,15]. However, other group 2a CoVs, including HCoV-OC43 and BCoV recognize N-acetyl-9-O-acetylneuraminic acid as a functional receptor [9]. While the cellular receptors for both groups 1 and 2 CoVs have been identified and independently confirmed, group 3 CoV receptors remains undetermined.

The avian CoVs, such as turkey CoV and infectious bronchitis viruses (IBV), have been classified in group 3, with IBV the most extensively studied. Recently, Winter and colleagues suggested that sialic acids are responsible for IBV strain Massachusetts 41 entry [16]. However, group 3 CoVs lack HE as a key viral protein regulating sialic acid binding, and the use of sialic acid would not explain the dependence on chicken cells for infection. Therefore IBV is unlikely to use sialic acids as a functional entry receptor, but rather as a non-specific attachment factor. Heparan sulfate may also serve as an attachment factor for the IBV strain Beaudette (IBV_Bdtt) [17]. IBV_Bdtt is a highly chicken embryo-adapted strain [18,19], which has an extensive tropism in cell culture and efficiently infects various cell types, including BHK-21 cells [17,19,20]. In contrast, clinical isolates and field strains of IBV typically only infect chicken cells.

In the effort to identify the receptor for group 3 CoVs, feline APN (fAPN) was reported to allow entry of the IBV strain Arkansas 99 (IBV_Ark99) [21]. This could therefore be the first indication of a more universal receptor for the CoV family. APN belongs to a family of metalloproteases [22]. It is a type II membrane-bound glycoprotein, and it is expressed on a variety of cell types, including granulocytes, monocytes, and fibroblasts. APN can also be found on the synaptic membranes of the central nervous system neurons, and on epithelial cells in the proximal convoluted tubules, intestinal brush border, and respiratory tract [11,23]. For coronaviruses in general, there is a cross-species restriction that permits cells of a certain species to be infected only by its own complimentary CoV. However, several studies on FIPV and HCoV-229E, CCV and TGEV have identified fAPN as a universal entry receptor for group 1 coronaviruses [11-13,23]. The demonstration that fAPN can allow infection by the IBV strain Ark_99 prompted us to examine both prototype and field isolates of IBV and test them for the potential use of fAPN as a receptor.

In this study, we first verified the use of the expressed fAPN as a receptor for FIPV and TGEV by transient and constitutive expression of fAPN in non-permissive BHK-21 cells. We also cultured seven strains of IBV, including Arkansas 99, Arkansas_DPI, California 99, Connecticut 46, Holland 52, Iowa 97, and Massachusetts 41 (designated as Ark99, Ark_DPI, CA99, Conn46, H52, Iowa97, and Mass41) as candidates to test for fAPN utilization by group 3 avian CoVs. Surprisingly, expression of fAPN did not increase viral infection in any of the strains tested. As a consequence, we conclude that fAPN is not a functional receptor during IBV entry. The authentic receptor is still under investigation.

## Results

**Figure 1:**
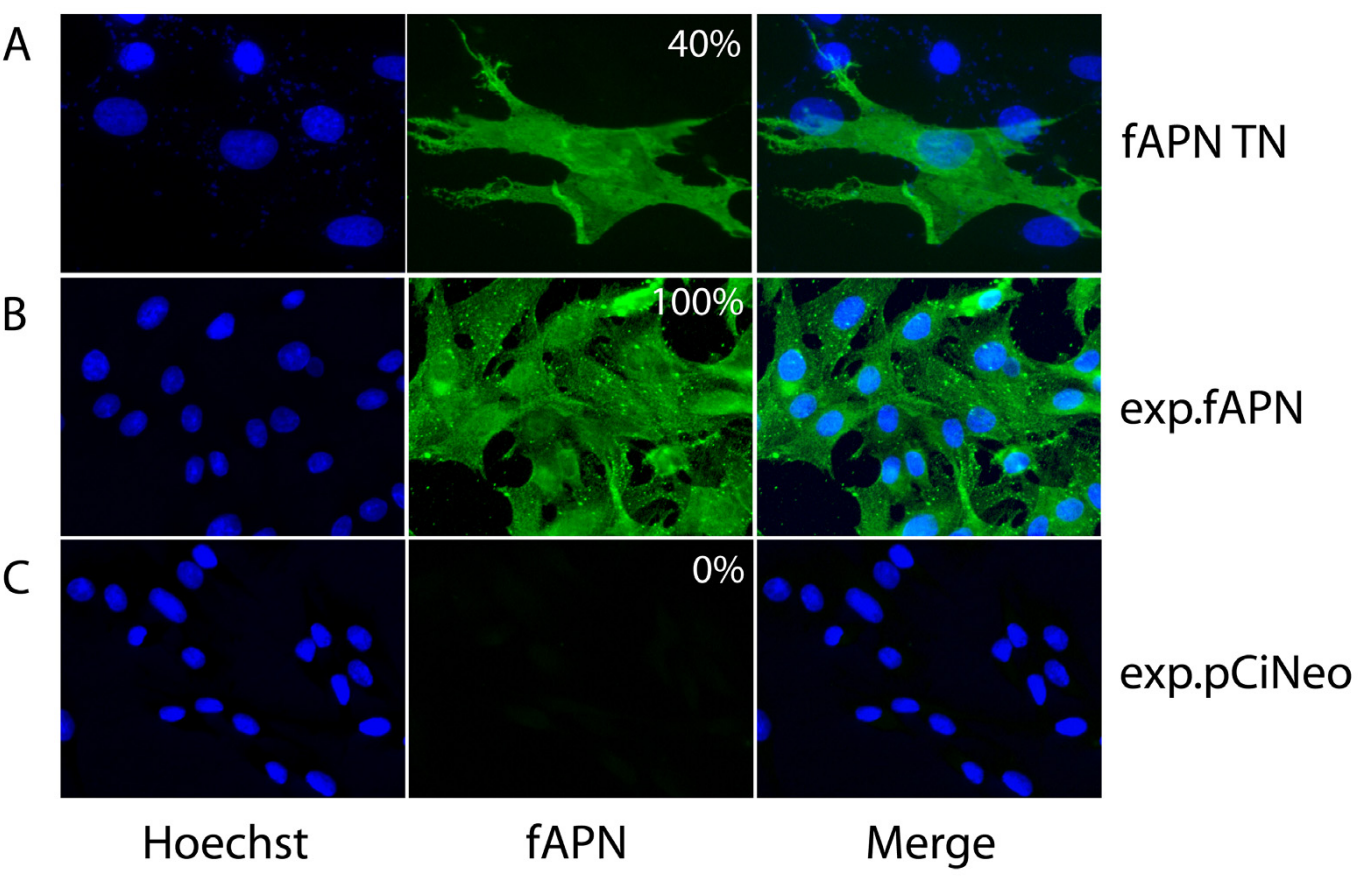
**fAPN is efficiently expressed on BHK-21 cell surface via transient or constitutive expression**. A) BHK-21 cells were transfected with fAPN/pcDNA3.1D/TOPO for 24 h and fAPN expression was stained with R-G-4 antibody post transfection. Transfection frequency was determined by counting 300 cells. B & C) BHK cell lines constitutively expressing fAPN and pCiNeo (vector only) were labeled with anti-fAPN monoclonal antibody R-G-4. fAPN expression frequency was determined by counting >300 cells.

In order to determine fAPN receptor usage by IBV, our goal was to rescue IBV infection in non-permissive cells by expressing fAPN on the cell surface. To authenticate fAPN expression, we first transiently transfected BHK-21 cells with fAPN/pcDNA3.1D/TOPO plasmid DNA for 24 hours. Samples were then subjected to immunofluorescence staining to detect fAPN protein expression (Fig. 1A). We found that nearly 40% of the BHK-21 population were efficiently transfected and stained positive for fAPN expression. Next, we verified the fAPN expression on the BHKexp.fAPN cell line that constitutively expresses fAPN and its negative control cells, BHKexp.pCiNeo (Fig. 1B and 1C). 100% of BHKexp.fAPN cells also stained positive, while the negative control showed no fAPN expression (Fig. 1).

**Figure 2:**
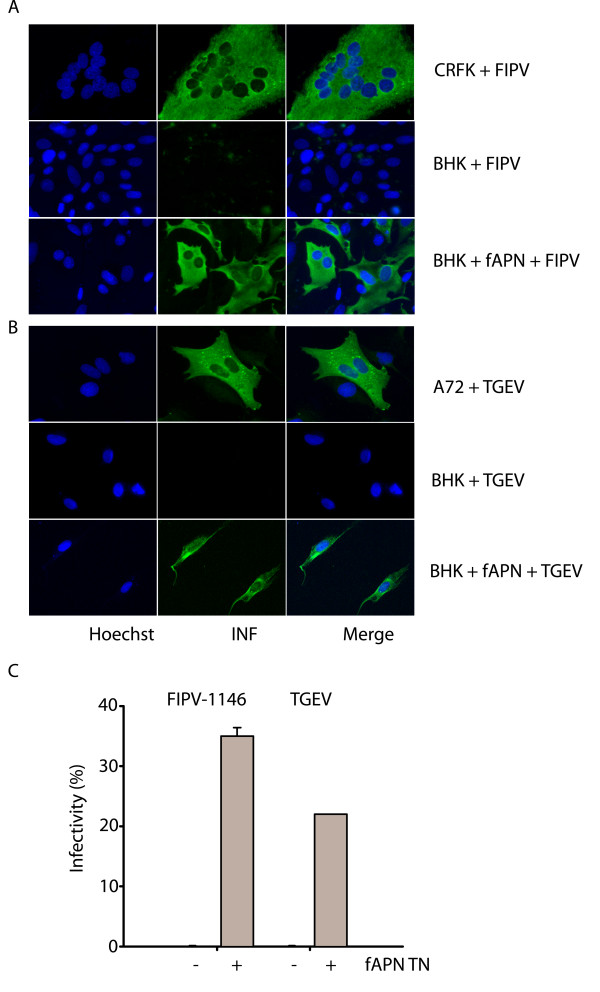
**fAPN expression rescues FIPV-1146 and TGEV infection in non-permissive BHK-21 cells**. A) CRFK, BHK, or BHK cells transfected with fAPN/pcDNA3.1D/TOPO were separately infected with FIPV-1146 for 9 h. Monolayers were stained with 17B71 monoclonal antibody to detect FIPV-1146 infections. B) A72, BHK, or BHK cells transfected with fAPN/pcDNA3.1D/TOPO were separated infected with TGEV for 8 h. Monolayers were labeled with rabbit polyclonal antibody 367 to detect TGEV infections. C) BHK and BHK transfected with fAPN/pcDNA3.1D/TOPO cells were separately infected with FIPV-1146 or TGEV. Viral infectivity was determined by counting >300 cells in three separate experiments. Error bars represent the standard deviation from the mean.

To verify the functionality of fAPN as a coronavirus receptor, we first tested its ability to rescue FIPV-1146 and TGEV infection of non-permissive cells, as reported in previous studies [11]. Feline kidney CRFK cells and canine fibroblast A72 cells are able to support FIPV-1146 and TGEV infection respectively (Dr. Edward Dubovi, Cornell University, personal communication). Therefore, these cell lines served as positive controls for FIPV-1146 and TGEV infection. We observed that FIPV-1146 and TGEV efficiently infected CRFK and A72 host cells respectively, but they were unable to mediate infection in BHK-21 cells (Fig. 2A and 2B), presumably due the lack of a functional receptor on the cell surface. However, transient transfection of fAPN in BHK-21 cells rescued FIPV and TGEV infection to 34% and 21% respectively (Fig. 2C). We therefore confirm that fAPN can function as an entry receptor for both FIPV-1146 and TGEV.

**Figure 3:**
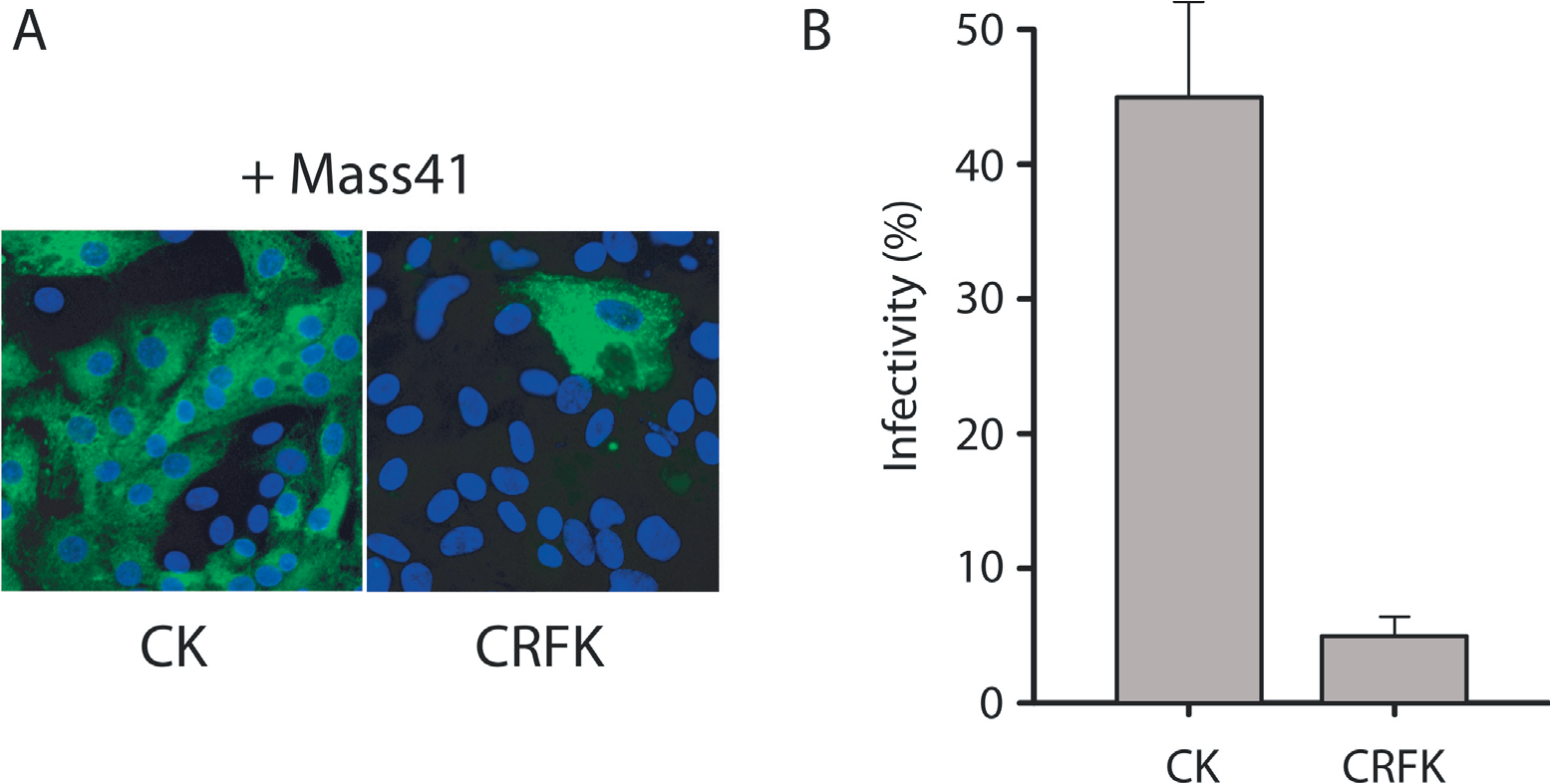
**IBV strain Mass41 can infect feline cells at low levels**. A & B) Primary CK or CRFK, cells were infected with IBV_Mass41 for 12 h and labeled with monoclonal α-S1(15:88) antibody to detect viral infection in three independent experiments. Viral infectivity was determined by counting >300 cells. Error bars represent the standard deviation from the mean.

Group 3 CoVs such as infectious bronchitis viruses (IBV) can infect primary chicken kidney cells at high efficiency, but they generally restrict interspecies infections [19]. However, previous studies have shown that feline kidney cells (FEK) can support IBV_Ark99 infection [21], suggesting the presence of a feline receptor on the FEK cell surface that enables IBV_Ark99 entry. Since IBV_Mass41 has served as a prototype virus to study CoV entry [24,25], we first examined the potential utilization of a feline receptor by IBV_Mass41. We found that whereas BHK-21 cells were resistant to IBV_Mass41 infection, and IBV_Mass41 infected CK cells at 45% efficiency, IBV_Mass41 was able to also infect feline CRFK cells at 6% efficiency (Fig 3B). As indicated by Miguel et al. [21], the IBV_Mass41 infection found in CRFK cells suggests the potential use fAPN as a receptor.

**Figure 4:**
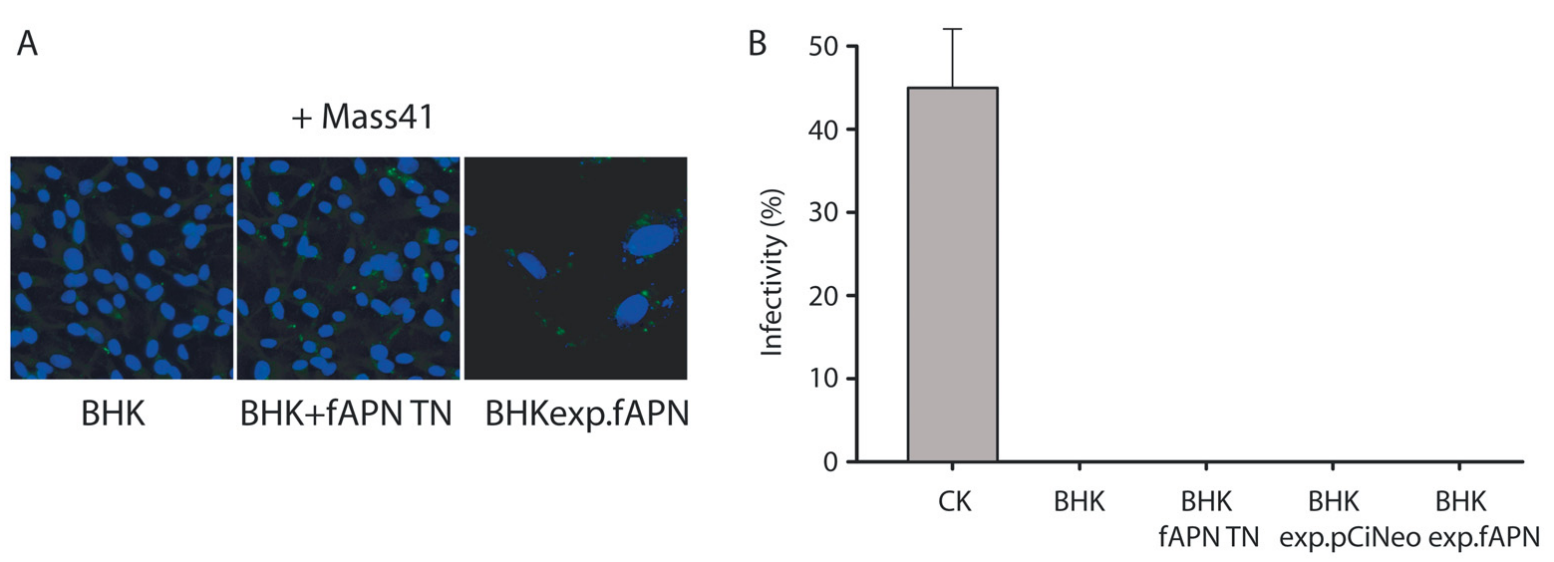
**IBV strain Mass41 does not utilize fAPN as an entry receptor**. A & B) Primary CK cells, wild type BHK cells, BHK cells transfected with fAPN/pcDNA3.1D/TOPO, BHK cells constitutively expressing fAPN cells, or control BHK cell lines, (BHK exp.fAPN and BHK exp.pCiNeo) were separately infected with IBV_Mass41 for 12 h and labeled with monoclonal α-S1(15:88) antibody to detect viral infection in three independent experiments. Viral infectivity was determined by counting >300 cells. Error bars represent the standard deviation from the mean.

To determine the potential role of fAPN as a receptor for the prototype IBV (Mass_41), we transiently transfected fAPN into BHK-21 cells. Both untransfected and fAPN-transfected BHK-21 cells failed to show any detectable infection with IBV_Mass41, whereas primary CK cells were efficiently infected (Fig. 4). To rule out the potential inhibitory effect of viral infectivity caused by transient transfection, we also examined IBV infection in cell lines constitutively expressing fAPN. We found that expression of fAPN in BHKexp.fAPN cells could not rescue IBV_Mass41 infection (Fig. 4A and 4B).

**Figure 5:**
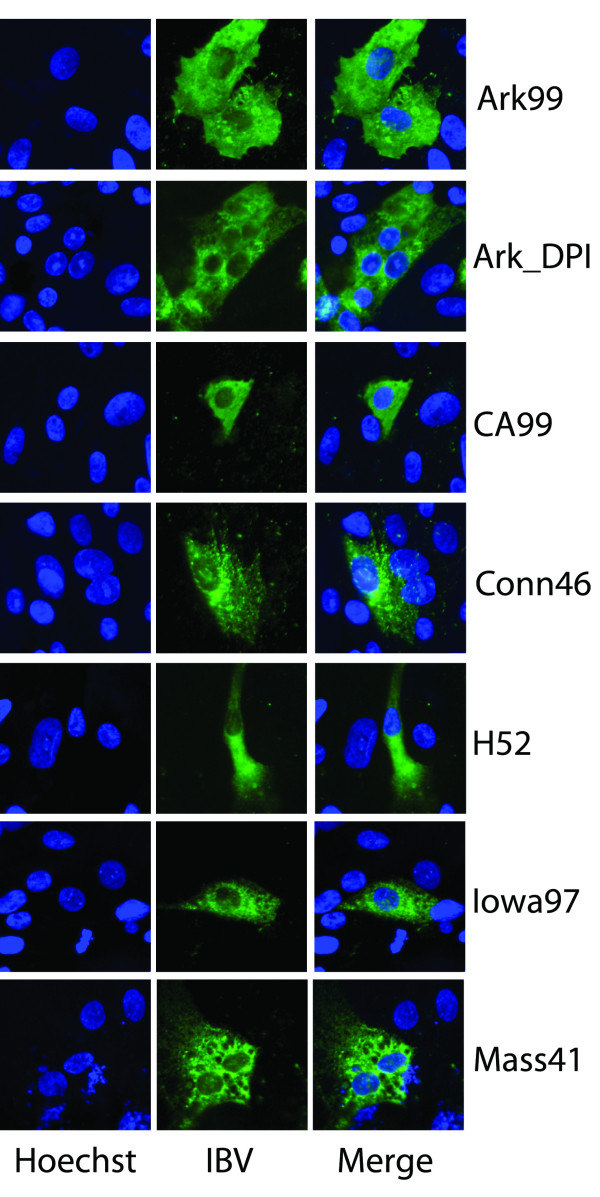
**IBV field isolates efficiently infect primary chicken kidney cells**. Monolayers of CK cells were separately infected with IBV strain Ark99, Ark_DPI, CA99, Conn46, H52, Iowa97, and Mass41 for 12 h. Cell monolayers were labeled with monoclonal α-S1 or M (15:88, 13:18, or 9:19) antibodies to detect viral infection.

**Figure 6:**
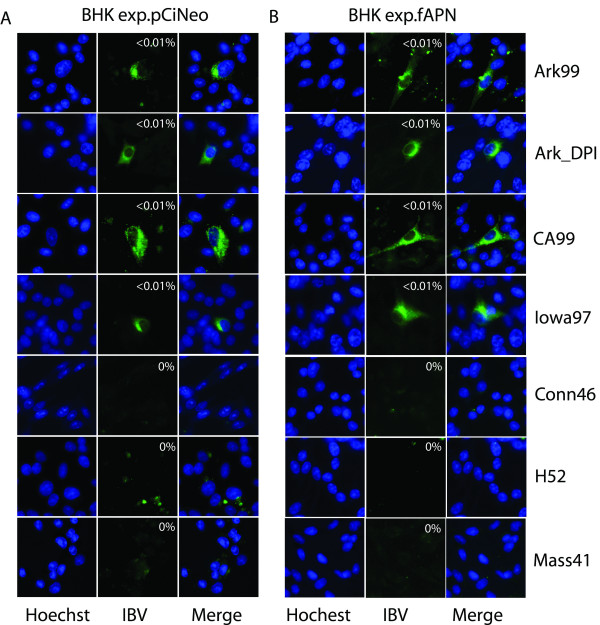
**fAPN is not a functional receptor for field strains of IBV**. BHKexp.pCiNeo (A) or BHKexp.fAPN (B) cells were independently infected with IBV strain Ark99, Ark_DPI, CA99, Iowa97, Conn46, H52, and Mass41 for 12 h. Cell monolayers were labeled with monoclonal α-S1 or M (15:88, 13:18, or 9:19) antibodies to detect and determine viral infections. Viral infectivity was determined by counting approximately 10^6 ^cells in three separate experiments.

To examine if fAPN might act in a strain specific manner, seven field IBV isolates: Ark99, Ark_DPI, CA99, Conn46, H52, Iowa97, and Mass41 were obtained and cultured in 10-day old specific pathogen free (SPF) chicken embryonic eggs. To verify virus infectivity, monolayers of CKC were individually incubated with all seven strains of IBV for 12 h. Figure 5 shows that CKC monolayers were found to be efficiently infected by all seven field strains of IBV tested. To determine the role of fAPN as a functional receptor for entry of these seven fields strains of IBV, as well as the prototype Mass 41, were inoculated onto BHKexp.pCiNeo or BHKexp.fAPN cell monolayers in triplicate. We found that BHK cells expressing empty vector alone (BHKexp.pCiNeo) were completely resistant to infection by IBV strains Conn46, H52 and Mass41. However, IBV strains Ark99, Ark_DPI, CA99, and Iowa97 showed limited infection in the same cells, with a level of infection of less than 0.01% (Fig. 6A). However, fAPN expression did not significantly enhance IBV infectivity in any of the virus strains tested (Fig. 6B). Consequently, our data show that IBV does not utilize fAPN as a functional receptor during virus entry.

## Discussion

Viruses make use of a variety of receptors to gain entry into their target cells. The ability to recognize sialic acids that are ubiquitously present on the cell surface gives influenza viruses the ability to indiscriminately infect varied tissue or cell types. In contrast, retroviruses require more specialized receptor interactions between viral glycoprotein 120 (gp120) and CD4 as well as other chemokine receptors on the T-helper lymphocytes during invasion [26]. Despite some subtle differences among different species or strains of viruses in the same family, they may still retain a trace of evolutionary similarity in terms of receptor utilization. However, receptor usage appears to have very little consensus among coronaviruses across different groups and species. Coronaviruses seem to be able to devise various strategies to interact with specific target host at the level of receptor binding.

In order to gain a more comprehensive understanding of IBV receptor utilization, we cultured several clinical strains of IBV for this study. Surprisingly, we found that the BHK-21 cells are in fact, weakly permissive to IBV_Ark99, IBV_DPI, IBV_CA99, and IBV_Iowa97 infections. However, fAPN expression on BHK-21 cell surface did not increase viral infectivity for any IBV strain. We therefore conclude that fAPN could not be a functional receptor for IBV entry. It is important to note that one, highly chick-embryo- adapted, IBV strain (IBV Beaudette) gives efficient infection in BHK cells [20]; combined with the data presented here, that a low level of infection of BHK cells could be obtained with some clinical strains, this suggests that there are no post-entry restrictions to IBV replication and gene expression that might account for a lack of infection of BHK cells.

How do we explain the discrepancy between these data and previous studies that show that fAPN is the receptor for IBV_Ark99 entry? According to our observations, IBV_Ark99 viruses were able to infect BHK-21 cells to a limited degree (<0.01%), although the infection profile was not always consistent among samples. The relevance of this very limited infection of hamster cells is unclear. As a consequence, previous studies may have shown only localized and under-represented populations of cells, which were infected by IBV_Ark99, since there was no further quantification of viral infectivity. In contrast, our present study was performed in a quantitative manner by measuring infection frequency of nearly 10^6 ^cells and we provide numerical virus infection data between fAPN-expressing and non-expressing cells.

Various studies have shown several potential receptor candidates for group 3 coronaviruses, which include fAPN, sialic acids, and heparan sulfate [16,21]. However, there is still little consensus on which of these might be the functional entry receptor *in vivo*. Sialic acid utilization as a functional entry receptor by IBV remains controversial since IBV generally has a highly restricted species tropism, and does not possess a HE glycoprotein that would normally be responsible for sialic acid destruction and hence efficient virus spread. Unlike influenza viruses that are established to use cell surface sialic acids for entry into a wide variety different cells, IBV is generally considered only to infect cells of chicken origin. The infected chickens often show lesions or lymphocyte infiltration in the ciliated epithelia cells, mucus secreting cells, and sub-epithelial cells along the respiratory tract [27]. IBV also infects lung, ovary, and kidney tissues [24]. In cell culture, IBV_Mass41 infects CKC but not chick embryo fibroblast cells or the chicken DF-1 fibroblast cell line (data not shown), suggesting possible cell type or tissue specificity for IBV infection.

Feline APN has been previously reported to serve as a functional receptor for IBV_Ark99 entry, a conclusion based in part on the ability of the virus to infect feline kidney cells (CRFK, also known as FEK) [21]. We have independently confirmed the ability of feline kidney cells to support IBV_Mass41 infection (Fig. 3A) with relatively high efficiency. As a consequence, cats could potentially serves as an intermediate host during an extension of coronavirus tissue tropism. In the case of SARS-CoV, viral transmission is thought to originate from Chinese horseshoe bats to Himalayan palm civet cats before jumping to humans [5,6]. The use of a feline receptor for IBV could potentially be another example of coronaviruses using feline cells as an interspecies reservoir to cross species boundaries. The reason why cats may serve as such an intermediate host is unclear, although it may be noteworthy that the endogenous feline coronavirus (FECV) can undergo distinct recombination events within feline cells that result in the emergence of the clinically different virus FIPV [28]. Also of potential relevance is the suggestion that the SARS-CoV S gene may be a mosaic of feline and avian sequences [29].

## Conclusion

We conclude here that IBV cannot utilize fAPN as a functional receptor. It is however important to note that although IBV does not utilize fAPN as an entry receptor, there is still a possibility that chicken APN (cAPN) may serve as the key receptor. Future work will address the potential usage of cAPN, as well as other chicken cell-specific proteins, as IBV receptors.

## Methods

### Viruses and cells

IBV Conn46, Iowa97, H52, and Mass41 were obtained from Dr. Benjamin Lucio-Martinez, Unit of Avian Health, Cornell University, and IBV Ark_DPI was obtained from Dr. Mark W. Jackwood, University of Georgia. IBV Ark99 and CA99 were obtained Dr. Shankar P. Mondal (University of California, Davis). Viruses were propagated in 11 day-old embryonated chicken eggs and purified as described previously [20]. Virus stocks were titered by infection of primary chicken kidney cells, followed by immunofluorescence microcopy with monoclonal anti-S antibodies after 8–12 h of infection. Typically, the highest titer stocks were able to infect between 50% and 70% of the CK cells present, and were used at this concentration for all infections, unless otherwise indicated.

FIPV-1146 and TGEV were obtained from Dr. Edward J. Dubovi, Animal Health Diagnostic Center, Cornell University, and were propagated in CRFK (feline kidney cell) and A72 (canine fibroblast cells) respectively. BHK-21 cells were purchased from ATCC and propagated twice weekly in DMEM, supplemented with 10% fetal bovine serum (FBS) 1% penicillin-streptomycin, and 10 mM HEPES (Cellgro). CRFK and A72 were grown in L:M media. BHKexp.fAPN or and BHKexp.pCiNeo cells were kindly provided by Dr. Kathryn V. Holmes (University of Colorado Health Sciences) and grown in 10% DMEM + 300 μg/ml G418 (Sigma).

Primary chicken kidney cells (CKC) were prepared as follows: in short, SPF White Leghorn Chicks (11–14 days) were sacrificed in a CO_2 _chamber, and kidneys were removed via aseptic techniques. Kidney cells were washed in 50 ml sterile phosphate buffer saline (PBS) twice via gentle stirring to remove red blood cells. The cells were then treated with 25 ml of trypsin-EDTA (Cellgro) for two minutes to separate large chunks of kidney tissues. Cell supernatant was mixed with 1 ml of FBS and centrifuged at 1,000 rpm for 2 min. Media were removed and kidney cells were resuspended in 25 ml of M20 media. Cell population was determined on a hemocytometer. For plating, 1–1.5 × 10^6 ^cell/ml was prepared in M25 media (M20 media with 5% FBS) and incubated in 5% CO_2 _at 37°C. For infection, 60% confluent cell monolayers were inoculated with virus stocks and incubated for 8, 12, or 24 hours before fixation.

### Plasmids and transfections

fAPN/pcDNA3.1D/TOPO and hAPN/pCiNeo plasmid DNAs were provided by Dr. Kathryn V. Holmes (University of Colorado Health Sciences). For standard transfection, 0.6 μg of plasmid DNA were premixed with 2 μl of Lipofectamine 2000 (Invitrogen) in 300 μl of Opti-MEM (Gibco) at room temperature according to manufacture's instructions. Cell monolayers were transfected at 37°C for 24 hours before fixation or virus infection.

### Virus infection and immunofluorescence microscopy

Infection and immunofluorescence microscopy were essentially performed as described previously [30] except with methanol fixation for detecting viral antigens. For fAPN staining, cell monolayers were fixed in 3% paraformaldehyde at room temperature and labeled with monoclonal antibody R-G-4 (provided by Dr. Tsutomu Hohdatsu, Kitasato University, Towada, Aomori, Japan). For viral antigen staining, anti-S1 monoclonal antibody 15:88 was used for IBV Massachusetts 41 and Iowa 97. The anti-S1 monoclonal antibody 13:18 was used for IBV Arkansas_DPI. The anti-M monoclonal antibody 9:19 was used for IBV Arkansas 99, Connecticut 46, California 99, and Holland 52. Monoclonal antibody 17B71 and rabbit polyclonal antibody 367 were provided by Dr. Edward Dubovi, Cornell University, and were used detect FIPV and TGEV infection respectively. The secondary antibodies Alex-fluor 488 goat anti-mouse IgG and Alex-fluor 488 goat anti-rabbit IgG were purchased from Molecular Probes. Cell nuclei were counter-stained with Hoechst 33258 (Molecular Probes). Cells were viewed on a Nikon Eclipse E600 fluorescence microscope. Images were captured with a SPOT RT Monochrome camera and SPOT Advanced v.4.0.9 software and processed with Adobe Photoshop v.7.

## Competing interests

The author(s) declare that they have no competing interests.

## Authors' contributions

VC participated in the design of the study, carried out the experiments presented, and drafted the manuscript. LM, JM, TO and CH carried out preliminary experiments on which the manuscript is based. BB contributed to the conception and design of the experiments. GW conceived of the study, participated in its design and coordination, and wrote the final manuscript. All authors read and approved the final manuscript.
